# Desire for predictive testing for Alzheimer’s disease and impact on advance care planning: a cross-sectional study

**DOI:** 10.1186/s13195-016-0223-9

**Published:** 2016-12-13

**Authors:** Meera Sheffrin, Irena Stijacic Cenzer, Michael A. Steinman

**Affiliations:** 1 Section of Geriatric Medicine, Division of General Internal Medicine Disciplines, Stanford University School of Medicine, Stanford, CA USA; 2 Division of Geriatrics, University of California at San Francisco, San Francisco, CA USA; 3 The San Francisco VA Medical Center, 4150 Clement St, Box 181-G, San Francisco, CA 94121 USA

**Keywords:** Alzheimer’s disease, Advance care planning, Attitudes and knowledge, Predictive testing

## Abstract

**Background:**

It is unknown whether older adults in the United States would be willing to take a test predictive of future Alzheimer’s disease, or whether testing would change behavior. Using a nationally representative sample, we explored who would take a free and definitive test predictive of Alzheimer’s disease, and examined how using such a test may impact advance care planning.

**Methods:**

A cross-sectional study within the 2012 Health and Retirement Study of adults aged 65 years or older asked questions about a test predictive of Alzheimer’s disease (*N* = 874). Subjects were asked whether they would want to take a hypothetical free and definitive test predictive of future Alzheimer’s disease. Then, imagining they knew they would develop Alzheimer’s disease, subjects rated the chance of completing advance care planning activities from 0 to 100. We classified a score > 50 as being likely to complete that activity. We evaluated characteristics associated with willingness to take a test for Alzheimer’s disease, and how such a test would impact completing an advance directive and discussing health plans with loved ones.

**Results:**

Overall, 75% (*N* = 648) of the sample would take a free and definitive test predictive of Alzheimer’s disease. Older adults willing to take the test had similar race and educational levels to those who would not, but were more likely to be ≤75 years old (odds ratio 0.71 (95% CI 0.53–0.94)). Imagining they knew they would develop Alzheimer’s, 81% would be likely to complete an advance directive, although only 15% had done so already.

**Conclusions:**

In this nationally representative sample, 75% of older adults would take a free and definitive test predictive of Alzheimer’s disease. Many participants expressed intent to increase activities of advance care planning with this knowledge. This confirms high public interest in predictive testing for Alzheimer’s disease and suggests this may be an opportunity to engage patients in advance care planning discussions.

## Background

Currently there is much research underway to predict the development of future Alzheimer’s disease. Biomarkers, such as genetic tests and imaging techniques, may be a valuable method to accurately predict development of dementia before the onset of cognitive impairment. While there are no current treatments than can halt or meaningfully change the course of dementia, this predictive testing could provide an opportunity for patients and families to plan for the future.

Predictive tests for Alzheimer’s disease can take many forms, including apolipoprotein E genotype testing, positron emission tomography imaging for cerebral amyloid pathology, cerebrospinal fluid tests [1], or tests for other biomarkers individually or in combination. While a few prior studies have indicated that there is public interest in predictive tests for dementia [2–5], these tests are not currently available to the general public.

Even in the absence of highly effective treatment options, predictive tests for dementia may be useful to help patients and families prepare for decisions that need to be made in the future, including advance care planning. In Huntington’s disease, a progressive neurodegenerative disease for which there is no treatment or cure, genetic testing among affected families provides prognostic information for individuals, provides a feeling of personal control, and can be used to plan for the future [6]. Individuals may wish to prepare their family for the diagnosis of Alzheimer’s disease and cognitive decline, prepare financially, or complete advance directives [7]. Additionally, this may be an opportunity to engage patients in advance care planning at a time when they are already contemplating their future health, before the onset of cognitive impairment. Previous studies have shown that many older adults wish to complete advance directives, but only a small percentage have done so [8, 9]. Predictive tests for dementia may allow people to make lifestyle or behavior changes, such as exercise for weight loss or improving hypertension control, at a younger age when it may have a greater impact on cognition [10]. Additionally, even in the absence of a cure or treatment, testing can help individuals prepare themselves and their families for care decisions in the future.

However, little is known about how predictive tests for dementia could change future behavior of the general population. The REVEAL study, which examined the impact of genetic education, APOE-e4 testing, and a counseling program for adult children of Alzheimer’s disease patients [11], provides some important insights into future behavior. Researchers found changes in some behaviors (reported changes to long-term care insurance; and reported change in medication, diet, and exercise behaviors) [12] and no changes in other behaviors (reported changes to health, life, or disability insurance) [7] by APOE-e4 or disclosure status. However, questions surrounding testing for Alzheimer’s disease and resulting changes in advance care planning have not been asked in a nationally representative sample of the general public. Understanding these issues may be valuable as tests predictive of dementia are being developed, to guide their implementation when they become available, and to gauge the nation’s interest in such tests.

Using data from a large, nationally representative sample, we explored who would take a hypothetical free and definitive test predictive of Alzheimer’s disease, and examined how use of such a test may impact advance care planning.

## Methods

### Subjects

All adults aged 65 or older who participated in the Health and Retirement Study (HRS) 2012 Experimental Module 6 were included in this study. The HRS is a nationally representative sample of community-dwelling older adults in the United States who are followed longitudinally and surveyed every 2 years. During every cycle a random subset of participants are asked additional questions in an experimental module, in addition to the core questions asked of all participants.

### Measures

Data for the study included questions from the HRS 2012 Experimental Module 6, and were linked to data from the HRS 2012 core module.

We examined characteristics of subjects who would and would not choose to be tested, including demographics, physical functioning, and comorbid conditions. We also examined subjects’ self-perceived memory and health, and self-perceived risk of developing Alzheimer’s disease.

Demographic characteristics included age, race, marital status, and educational level. We assessed physical functioning as the total number of activity of daily living (ADL) difficulties and dependencies based on self-reported abilities on six ADL domains. We assessed comorbid conditions as the self-reported presence or absence of seven common conditions in older adults (hypertension, diabetes, lung disease, heart disease, cancer, arthritis, and stroke). Subjects rated their health status in categories of excellent, very good, good, fair, or poor. We classified those answering fair or poor as having poor self-perceived health. Self-perceived memory was rated and classified similarly. Subjects rated their perceived chance of developing Alzheimer’s disease in the future on a scale from 1 to 100. We classified a score ≤ 25 as low self-perceived risk of Alzheimer’s disease, informed by the frequency distribution of this variable in which most people clustered in the midpoint and very low and very high ends of the scale.

We examined two main outcomes: willingness to take a test predictive of future Alzheimer’s disease; and likelihood of completing an advance directive or living will if they knew they would develop Alzheimer’s disease. For the first outcome, subjects were asked: “If you could receive a test from your doctor, free of charge, that would definitely determine whether or not you would develop Alzheimer’s disease in the future, would you want to be tested?” Answer choices included yes, no, do not know, and refuse to answer.

For the second outcome, subjects were told to imagine they knew they would develop Alzheimer’s disease in the future, and with this knowledge to rate the chance of completing advance care planning activities. They were asked: “If you knew you would develop Alzheimer’s disease in the future, how likely (where 0 means no chance and 100 means absolutely certain) would you be to set up an advance directive or living will to let family members and doctors understand how you wish your health care to be managed?”

We also examined a secondary outcome of likelihood of discussing health and medical plans with loved ones if they knew they would develop Alzheimer’s disease. On a similarly worded question, subjects rated their likelihood to discuss their health and medical plans with loved ones. Answers were on a scale from 0 to 100; we classified scores > 50 as being likely to complete that activity. This cutoff point was chosen because a score of 50 and above indicated participants would be more likely than not to complete that activity.

### Analysis

Health and Retirement Study participants are selected using a complex sampling design involving clustering, stratification, and oversampling of certain segments of the population. We used methods as recommended by HRS investigators [13] to adjust for these survey design features and generate nationally representative estimates. We used survey-appropriate measures of association to assess how subject characteristics impacted with willingness to take a test for Alzheimer’s disease, and completing an advance directive and discussing health plans with loved ones. In the bivariate analyses, the cutoff points for age, self-rated health, and self-rated memory were prespecified. Because of the complex survey weighting, the raw total numbers reported in the tables may not precisely match their corresponding percentages, because those percentages are adjusted for the survey design.

The Research Committee at the San Francisco VA Medical Center approved this research. The USCF Committee on Human Research exempted this study from review.

## Results

Among the 874 individuals selected for participation in this substudy, 861 (99%) answered the question about a test for Alzheimer’s disease. All of the participants in the substudy were already participating in the larger HRS study, which administers surveys to respondents every 2 years. In this substudy, mean age was 74 years and 56% were female (Table 1). Overall, 75% (*N* = 648) of respondents stated they would take a free and definitive test predictive of Alzheimer’s disease.

**Table 1:** Subject characteristics

Characteristic	Total (*N* = 861)
Age (years), mean ± SD	74.2 ± 7.2
Female	506 (56.0%)
Race	
White	665 (83.0%)
African American	113 (7.9%)
Latino	63 (6.4%)
Other	20 (2.8%)
Education less than high school	188 (18.8%)
Married or partnered	499 (59.5%)
Number of ADL difficulties	
0 difficulties	687 (82.0%)
1 difficulty	86 (9.8%)
2+ difficulties	88 (8.2%)
Number of ADL dependencies	
0 dependencies	777 (90.2%)
1 dependency	42 (4.9%)
2+ dependencies	42 (4.9%)
Number of comorbidities	2.4 ± 1.3
Poor self-rated health	256 (27.8%)
Poor self-rated memory	296 (33.4%)
High self-rated risk of Alzheimer’s disease	439 (50.6%)
Already completed an advance directive	119 (14.5%)

Data presented as *n* (%) unless stated otherwise

*ADL* activity of daily living

Older adults willing to take the test had similar race and educational levels to those who would not, but were more likely to be ≤75 years old (adjusted odds ratio 0.71 (95% CI 0.53– 0.94) compared with those >75 years old) and less likely to have completed an advance directive (adjusted odds ratio 0.56 (95% CI 0.33–0.92)) (Table 2). By means of context, after adjusting for demographics and other factors, older adults who had already completed an advance directive had a predicted probability of 77% (95% CI 73–81%) of being willing to take a test for Alzheimer’s disease, compared with 65% (54–77%) in those who had not completed an advance directive (not shown in table). There were no differences in willingness to take the test by level of self-perceived health or memory problems, self-perceived risk of Alzheimer’s disease, ADL difficulties or dependencies, or number of comorbidities.

**Table 2:** Bivariate and multivariable predictors of wanting to take a test for Alzheimer’s disease

Characteristic	Bivariate analyses		Multivariable analyses
	*n* (%) desiring test for AD	*P* value	odds ratio (95% CI)
Age			
≤ ﻿75 years	378 (78.4%)	0.003	Referent
> 75 years	270 (69.6%)		0.71 (0.53–0.94)
Sex			
Male	269 (76.0%)	0.568	Referent
Female	379 (74.0%)		0.99 (0.7–1.39)
Race			
Nonwhite	159 (79.7%)	0.205	Referent
White	489 (73.9%)		0.75 (0.41–1.38)
Education			
More than high school	510 (78.9%)	0.266	Referent
Less than high school	138 (70.7%)		0.75 (0.47–1.19)
Marital status			
Single	265 (71.9%)	0.136	Referent
Married or partnered	383 (77.0%)		1.17 (0.8–1.72)
Number of ADL difficulties			
0 difficulties	521 (75.0%)	0.981	Referent
1 difficulty	65 (75.0%)		0.98 (0.52–1.84)
2+ difficulties	62 (74.1%)		0.79 (0.46–1.35)
Number of comorbidities			1.11 (0.94–1.32)
Self-rated health			
Good/excellent	454 (74.1%)	0.439	Referent
Fair/poor	193 (76.9%)		1.18 (0.76–1.81)
Self-rated memory			
Good/excellent	433 (76.6%)	0.253	Referent
Fair/poor	214 (71.5%)		0.72 (0.44–1.17)
Self-rated risk of AD			
Low	303 (72.0%)	0.110	Referent
High	345 (77.7%)		1.33 (0.9–1.97)
Advance directive completed			
No	569 (76.7%)	0.029	Referent
Yes	79 (64.4%)		0.56 (0.33–0.92)

*AD* Alzheimer’s disease, *ADL* activity of daily living, *CI* confidence interval

Next, subjects were asked about their intended behaviors if they learned with certainty that they would develop Alzheimer’s disease. In this setting, 87% reported they would be likely to discuss health plans with loved ones. Most respondents (81%) reported they would be likely to complete an advance directive, although overall only 15% reported having done so already.

## Discussion

In this nationally representative study of 874 community-dwelling older adults, 75% were interested in a hypothetical test that would predict development of Alzheimer’s disease in the future. This high desire did not differ by sex, race, functional status, comorbidity, perceived memory, or perceived risk of Alzheimer’s disease. Additionally, in the face of a positive test, 87% reported they would be likely to discuss health plans with loved ones, and 81% reported they would be likely to complete an advance directive.

This degree of public interest in predictive testing for dementia is similar to what has been seen in several prior online and telephone studies with nonrandom samples [2, 4, 5, 14]. In a 2014 online survey of subjects enrolled in an online community interested in Alzheimer’s disease prevention research [4], 81% wanted genetic testing for Alzheimer’s disease if paid by insurance, and 70% thought genetic testing was important even in the absence of any effective intervention. In a telephone survey of 2678 subjects across five countries done in 2013 [2], 67% of all subjects stated they would be somewhat or very likely to obtain testing if it was available in the future. In an additional analysis, knowing Alzheimer’s disease is a fatal condition did not affect the results. In related work, Roberts [15] used nationally representative data from the 2010 HRS to assess to what degree individuals aged 50 years and older wanted to know their chances of developing Alzheimer’s disease. They found that 60% of respondents age 50 years and older endorsed “somewhat” or “strong” agreement with the desire to know their future chances of getting this disease. While there are substantial differences between a general interest in risk and willingness to take a fully predictive test, these results are generally consistent with our findings.

Two prior studies of predictive testing for Alzheimer's disease asked questions regarding advance directives. In a small 2001 study asking a question similar to our own, out of 314 US adults responding to a random-digit-dialed telephone survey, 79% of would take a hypothetical perfect genetic test for Alzheimer’s disease [14]. Additionally, 84% stated that signing an advance directive was one of the actions they would take after a positive result. In a 2012 online survey of 772 respondents enrolled in a national online panel of US residents, 70–75% would take a test predictive for Alzheimer’s disease [5], and 51% would sign an advance directive document if receiving a positive test.

These studies of subjects with high interest in Alzheimer’s disease and responders to telephone and Internet surveys found similar results to those presented here. We found similar high interest in predictive testing for Alzheimer’s disease and intention to complete advance directives in our nationally representative sample of community-dwelling older adults. Our findings add to this existing body of knowledge in several important ways. Using a nationally representative sample helps to overcome the limitations of smaller or more selected samples, and confirms the high public concern and worry about Alzheimer’s disease as found in a prior study using the HRS [15]. The HRS is much less affected by response bias, utilizing careful weighting procedures to account for interview nonresponse. Additionally, our ability to evaluate a variety of potential predictors of attitudes toward testing helps to shed light on the factors that influence desire for predictive testing.

We did not find clinically meaningful differences in desire for predictive testing by level of comorbidity or disability, age, or perceived risk of Alzheimer’s disease. While somewhat surprising, if an individual’s threshold for testing is very low, patient characteristics may not affect the desire for testing and overall interest will be high.

Additionally, high interest in testing may reflect increasing media attention to the topic of dementia. There may be a general lack of knowledge of available treatments for dementia, perception that they are more effective than they actually are, or the hope that a treatment or cure will soon be available. This is supported by prior work which found that 40% of those surveyed thought prescription drugs that prevent Alzheimer’s disease are currently available [15]. Additionally, many believed there were behaviors that could be protective against Alzheimer’s disease, with 40% stating keeping physically active would be helpful and 20% believing taking vitamins/herbal supplements would help [15]. These beliefs may account for high interest in testing among all groups of respondents. Subjects may also desire predictive testing to aid in preparing family members for the development of Alzheimer’s disease [16].

Understanding the potential demand for predictive testing for Alzheimer’s disease may guide implementation of availability and use of these tests when they do become widely available. Disadvantages of large-scale predictive testing must be considered, including the false positives and need for education or counseling to assist patients in interpreting the results. Possible harms of predictive testing for Alzheimer’s disease could potentially include excess worry, employment discrimination, and ineligibility for long-term care insurance, among others. These possible harms of testing must be considered, especially given the current lack of effective prevention and the fatal nature of dementia. However, engaging patients who present to a physician seeking predictive testing for Alzheimer’s disease could be a unique opportunity to discuss advance care planning with older adults. This would allow them to make choices and express wishes for future care before they became cognitively impaired, and to initiate the conversation. Advance care planning is a process [17] and predictive testing for dementia may be an additional moment in which physicians can guide patients in the steps of determining, sharing, and documenting their values and preferences.

Our study does have some limitations. The question stem did not clearly include information on the progressive nature of Alzheimer’s disease or lack of very effective treatments, and it is possible that some subjects did not have this knowledge. However, there is increasing attention on Alzheimer’s disease in the media, and subjects may still be interested in a test because they have hope for future treatments or will use it to plan their future. We must note that the study question asked a hypothetical question about a free and definitive test for Alzheimer’s disease. A perfectly predictive test does not exist; all tests will have some rate of false positives and false negatives and no test is ever likely to be truly definitive. However, patients often assume tests are perfect and will yield definitive results, even when that is not the case. A clear and concise hypothetical question can be useful to assess the underlying preferences of the public regarding testing for Alzheimer’s disease in general; those underlying preferences would be modified based on the nuances, test characteristics, and cost of whatever test becomes available.

The expressed high desire for taking a hypothetical predictive test for Alzheimer’s disease may overestimate the demand for these tests once they are available, because people may not act on their current wishes. Studies of predictive testing for Huntington’s disease predicted uptake of 50–80%, although actual uptake was lower when testing was developed [6]. This may be due to more fears of stigmatization and genetic discrimination associated with Huntington’s disease [6], although there are more legal protections for the latter in recent years. Despite this, the high interest found by our study and others suggest that even if the percentage of subjects seeking testing is much lower, there will still be a substantial demand for predictive tests for Alzheimer’s disease. True levels of advance care planning may also be lower than what subjects reported they intend to do. However, the current rate of advance directive completion found in our study (15%) is very low, and discussing advance care planning with patients seeking predictive testing for Alzheimer’s disease is an opportunity to substantially improve the rates of discussions of advance care planning and completion of advance directives. Finally, the question stem describes a hypothetical free and definitive test predictive of Alzheimer’s disease, and some may argue that this will never exist. While a test is unlikely to be 100% definitive, research is being conducted to find a predictive test that is both highly sensitive and specific. Additionally, if the cost of testing is paid by insurers it may be provided to patients at low to no cost, and may seem “free” to them.

## Conclusions

In this large national sample of 874 community-dwelling older adults, 75% would take a free and definitive predictive test for Alzheimer’s disease. Additionally, if subjects knew they were likely to develop Alzheimer’s disease, 87% reported they would discuss future health plans with loved ones. This interest and the potential for high demand for predictive testing when it is available should be considered as these tests become available. Furthermore, our findings suggest that predictive tests for Alzheimer’s disease may provide an opportunity to engage older adults in activities of advance care planning.

## Abbreviations

ADL: Activity of daily living
HRS: Health and Retirement Study
